# Self-management of type 2 diabetes using a co-designed text based mobile health (mHealth) intervention in Nepal: A study protocol for randomised controlled trial

**DOI:** 10.1371/journal.pone.0335333

**Published:** 2025-11-04

**Authors:** Namuna Shrestha, Padam Simkhada, Om Kurmi, Shiva Gautam, Biraj Karmacharya, Akina Shrestha, Rajendra Tamrakar, Ak Narayan Poudel

**Affiliations:** 1 School of Human and Health Sciences, University of Huddersfield, Huddersfield, United Kingdom; 2 Centre for Healthcare and Communities, Coventry University, Coventry, United Kingdom; 3 Department of Public Health, Kathmandu University School of Medical Sciences, Dhulikhel, Nepal; 4 Dhulikhel Hospital, Kathmandu University Hospital, Dhulikhel, Nepal; University of Science and Technology of Fujairah, YEMEN

## Abstract

Mobile health (mHealth) is increasingly being used for managing chronic diseases proving its feasibility, acceptability and effectiveness in improving the health outcomes. However, effectiveness of mHealth intervention for self-management of Type 2 diabetes remains unexplored in the context of Nepal. This study aims to develop and evaluate the effectiveness of a co-designed text based mobile health intervention for self-management of Type 2 diabetes in Nepal. We will conduct a six month, two arm (1:1) parallel group randomised control trial involving 154 adults with Type 2 diabetes. The primary outcome includes change in haemoglobin A1c level at six months follow up. Secondary outcomes include change in self-management outcomes such as self-efficacy, perceived support, diabetes related stress, health related quality of life and self-care activities. Primary and secondary outcomes will be summarized descriptively, and comparisons will be made using chi-square tests for categorical data and independent sample t-tests for continuous data. The trial is registered at ClinicalTrials.gov (NCT06623006).

## Introduction

Diabetes is a growing public health challenge, particularly in low and middle income countries (LMICs) [1]. In 2024, 589 million people (20–79 years) were living with diabetes, expected to rise to 853 million by 2050 [2]. More than four in five adults with diabetes are living in LMICs [2]. In 2024, diabetes accounted for 3.4 million deaths [2] and remains a major cause of blindness, kidney failure, heart attack, stroke and lower limb amputation [1].

Following the global trend, the prevalence of diabetes is increasing in Nepal [3,4]. It was the ninth leading cause of death in 2021 and accounted for 2.6% of total deaths [5].The International Diabetes Federation (IDF) estimated a prevalence of 7.7% in 2024 with the proportion expected to rise to 9.2% by 2050 [6]. The escalating burden of disease may place serious challenges on the resource constrained national health system.

Access to care is of utmost importance for people with diabetes in the course of their disease; however, disparities exist across socio-economic and geographical distribution posing challenges in accessing the diabetes care services [7]. WHO STEPS survey Nepal 2019 reported that about three-fourths (73.5%) of the people were unaware of their raised blood sugar. Of those who were aware of their condition, about 6% were not on treatment and only 70% of adults with raised blood sugar reported adherence to medications [8]. Limited awareness among people have restricted their ability to self-manage their condition and increased the risk of complications [9]. Public health facilities in Nepal lack capacity to manage the rising burden of NCDs, including diabetes in terms of availability of qualified and trained health staff, equipment and essential medicines [10–12].

Self-management is essential for preventing and controlling the disease [1] and includes healthy coping, healthy eating, being active, taking medication, monitoring, reducing risks and problem solving [13] with support from family members, friends and relatives and in consultation with health service providers [14]. Self-management has proven to be cost effective through a reduction in hospital admissions and health care costs [15]. Besides, it has been shown to improve HbA1c level and has a positive effect on other clinical, psychosocial, and behavioural aspects of diabetes along with improvement in quality of life by reducing onset and/or advancement of diabetes complications [14,16].

Mobile health (mHealth) is becoming increasingly popular in the self-care of chronic diseases [17] and has proven to be a therapeutic strategy that could improve diabetes management despite their economic status [18–24]. In Nepal, about three-fourth (73%) of the households have at least some form of smartphone penetration and 37.9% have internet access [25].

In resource limited countries like Nepal, mHealth technology can be a cost-effective approach to delivering care and improving health outcomes. Evidence from Nepal have demonstrated the feasibility, acceptability [26,27] and even effectiveness [26] of mHealth in improving the health outcomes for chronic conditions like hypertension [26,27]. mHealth has also shown to be an effective means to communicate health related information and support positive behaviour change in a short period [28]. Despite the availability of a body of evidence on the acceptability of mHealth in improving service utilisation and health outcomes for chronic conditions, the application of behaviour change interventions through mHealth for self-management of diabetes is yet to be evaluated.

## Objectives

This study aims to evaluate the effectiveness and acceptability of a co-designed text based mHealth intervention that improves self-management in people with Type 2 diabetes at Dhulikhel Hospital of Nepal.

Objective 1: To determine the effectiveness of co-designed text based mHealth intervention in improving self-management (HbA1c level, self-efficacy, perceived support, diabetes related stress, health related quality of life and self-care behaviors) of Type 2 diabetes.

Objective 2: To determine the acceptability of a co-designed text based mHealth intervention in improving self-management among people with Type 2 diabetes.

## Materials and methods

**Fig 1:**
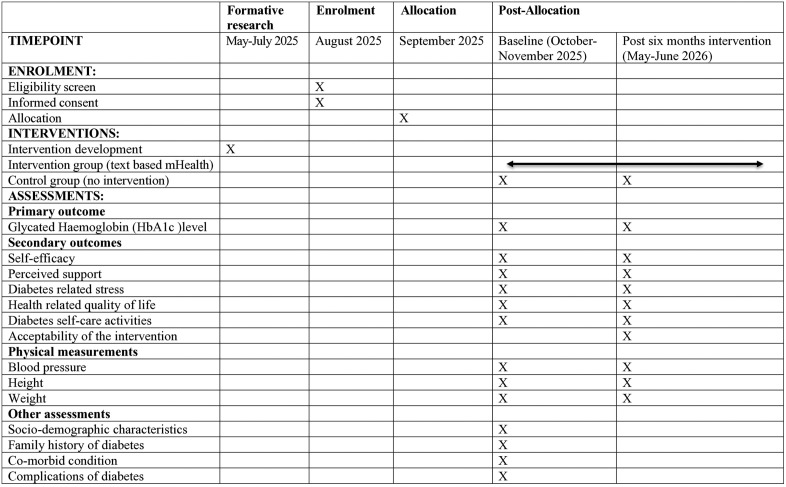
SPIRIT schedule of enrolment, interventions and assessments.

**Fig 2:**
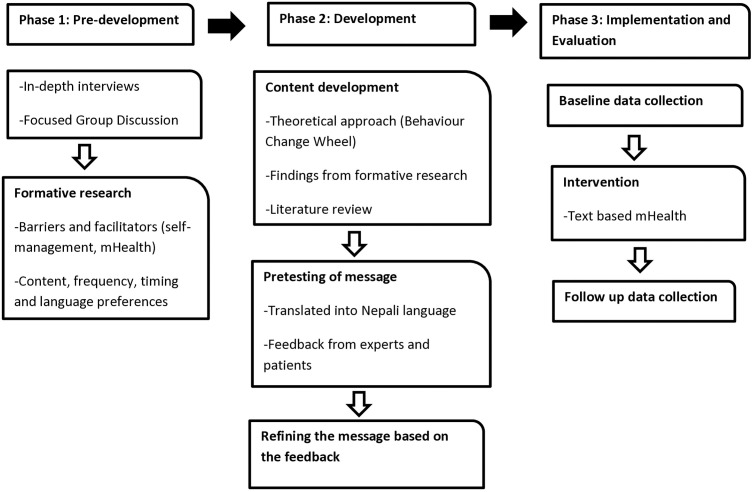
Methodological framework.

A Standard Protocol Items: Recommendations for Interventional Trials (SPIRIT) schedule for enrolment, intervention and assessment is presented in Fig 1. The methodological framework of this study is illustrated in Fig 2.

### Study design

This protocol describes a 6-month text-based mHealth intervention. It employs a two-arm parallel group randomised control trial to evaluate the effectiveness and acceptability of a co-designed text-based mHealth intervention in improving self-management among people with Type 2 diabetes in Dhulikhel Hospital, Kavrepalanchowk, Nepal. The protocol was developed in accordance with the SPIRIT 2013 statement [29] and Consolidated Standards of Reporting (CONSORT)-EHEALTH checklist [30].

### Study setting

The study will be conducted in Dhulikhel Hospital located in Kavrepalanchowk district of Nepal. It is a tertiary level hospital, providing comprehensive and quality health services to about 2.5 million people with a particular focus on rural and unserved populations [31].

### Study participants

#### Eligibility criteria.

Inclusion criteria for the study include a. adults aged 18 years and above b. clinical diagnosis of Type 2 diabetes c. participant who have a mobile phone and can read the message on their own or with the help of family members. Participants are not eligible if they are a. physically and mentally not able to provide consent b. female who are pregnant during the study period.

### Recruitment

All participants who meet the eligibility criteria will be invited to participate in the study. The participants will be approached by the lead researcher. Those who meet the inclusion criteria will be provided with the information sheet containing information about the study’s objective, procedures, duration, potential risk and benefits, confidentiality, their voluntary participation and frequency of participation in the study. The participants will be provided an opportunity to ask questions and get clarification. They will also be presented with a consent form to review and sign if they agree to participate.

### Intervention

#### Intervention development.

The intervention development will follow a systematic, theory based and evidence informed process based on three core components: The Behaviour Change Wheel (BCW) framework, formative qualitative study and insights from a targeted literature review.

#### Theoretical framework.

There is a growing recognition on the importance of incorporating theories of behaviour change in the design of interventions aimed at behaviour modifications [32,33]. A scoping review has identified 82 theories of behaviour change applied to design public health interventions [34]. Given the availability of multiple theories for health behavior change [34] and inability of a single theory to explain wide range of health behaviors [35], the need of comprehensive theory for behavior change is apparent.

Behaviour Change Wheel (BCW) has been developed as a comprehensive theory and evidence-based tool developed through expert consensus and validation. The BCW will serve as the primary theoretical framework guiding the intervention development in this study. At the core of the BCW lies the COM-B model which identifies Capability (C), Opportunity (O) and Motivation (M) as the key components influencing the behaviour. The process begins with a behavioural diagnosis-understanding of capability, opportunity and motivation to achieve the target behavior and what intervention functions and behaviour change techniques are likely to be effective to achieve the target behavior [35]. The BCW is used to design a number of behavior change interventions (36–40) including design of mHealth intervention for self-management of different forms of diabetes (41–44).

#### Literature review.

A targeted literature review will be conducted to identify effective behavior change strategies, key content themes, and messaging approaches that have demonstrated positive outcomes in previous mHealth interventions aimed at supporting self-management of Type 2 diabetes. The review will also examine elements of intervention design and delivery, including message frequency, language, tone, and timing. Findings from this review will be synthesised and mapped onto the BCW framework, ensuring that the intervention content is both theoretically grounded and evidence-informed.

#### Formative research.

In parallel, formative qualitative research will be conducted to explore the perspectives of both service providers and people living with Type 2 diabetes regarding the use of text-based mHealth interventions for supporting diabetes self-management. The study will aim to identify potential barriers and facilitators to the implementation of the intervention within the local context. Additionally, insights from this research will inform the development of the SMS content, including preferences around message frequency, timing, and language, ensuring that the messages are acceptable and relevant.

Drawing on the insights from the BCW, the literature review, and the formative research, a library of text messages will be developed in English language at first and then translated into Nepali language.

### Pre-testing

The draft text messages translated into Nepali language will be shared with experts to assess the relevance of the content based on their clinical expertise and experience with patients. Each expert (6–10) will be asked to rate each item on scale of 1–4 where, 1 = not relevant, 2 = somewhat relevant, 3 = quite relevant and 4 = highly relevant. Based on these ratings, a Content Validity Index (CVI) will be calculated [36]. Additionally, the messages will be pretested with a group of participants who are similar to the target population to evaluate their clarity, usefulness and relevance based on 5 point Likert scale (1 = strongly disagree to 5 = strongly agree). Furthermore, participants will also be asked to provide their additional feedback or suggestion to improve the content of message [37]. Feedback from both experts and participants will be used to refine the final set of message prior to implementation. The entire process will be conducted over a one-month period.

### Intervention

The intervention group will receive a co-designed text based mHealth intervention (text messages) developed in Nepali language in addition to standard care while the control group will receive only standard care.

### Standard care

Standard diabetes management and care provided at the visiting health facilities.

### Intervention delivery

A recognised IT company will be engaged to facilitate the delivery of text messages to the participants. For this, the provider will require access to phone numbers of the participants. A contract will be signed with the company to protect privacy of the participants and to ensure no sensitive information is disclosed.

### Study outcomes

The primary and secondary outcomes along with their measures and point of measurements are displayed in Table 1.

**Table 1:** Study outcomes.

Outcomes	Measures	Baseline (0 month)	Follow up (6 months)
**Primary Outcome**			
Glycated Haemoglobin (HbA1c)level	HbA1c test	✔	✔
**Secondary Outcomes**			
Self-efficacy	Diabetes Self Efficacy Scale, Self-Management Resources Center	✔	✔
Perceived support	Multidimensional Scale of Perceived Social Support	✔	✔
Diabetes related stress	Diabetes Distress Scale	✔	✔
Health related quality of life	WHOQOL- BREF	✔	✔
Diabetes self-care activities	Revised scale of Summary of Diabetes Self Care Activities measure	✔	✔
Acceptability of the intervention	Measured through “Yes” or “No” questions on usefulness, appropriateness, sharing, learning and recommendation to others including support provided in changing behavior.		✔

### Validity and reliability of the tools

Diabetes Self Efficacy Scale (DSES) is an 8-item with a scale ranging from 1 (not at all confident) to 10 (totally confident). The score for the scale is the mean of eight items where higher number indicated higher self-efficacy [38]. This scale is found to have good internal consistency and test-retest validity [39,40] and have been modified and used in Nepalese context [41].

The Multidimensional Scale of Perceived Social Support is a 12-item, 7-point Likert scale (1 as very strongly disagree to 7 as very strongly agree) to assess perceived support from family, friends and significant other [42]. The Nepalese version of this scale is found to have good construct validity and reliability [43].

The Diabetes Distress Scale consists of 17 questions in total: Emotional burden (5 questions), Physician related distress (4 questions), Regimen related distress (5 questions) and Interpersonal distress (3 questions). Responses to the question range from 1 (slight problem) to 6 (very serious problem). The mean is calculated for each subscale. This scale is found to be valid and reliable tool to assess distress among people with Type 2 diabetes in context similar to Nepal [44].

WHOQOL- BREF consists of 26 items across four domains: physical, psychological, social and environmental including questions on the overall quality of life and general health. The score ranges from 1 to 5 on a response scale. Higher scores denote higher quality of life [45]. This tool is found to have good internal consistency and validity in assessing the quality of life of people across different cultures [46–48] including Nepal [49].We will use Nepali version of WHOQOL-BREF.

The Revised Scale of Summary of Diabetes Self Care Activities measure consists of core set of 11 items from Summary of Diabetes Self Care Activities measure and an additional 14 questions [50]. It has been a valid and reliable tool for assessing self-care behaviour among adults with diabetes [51].

The acceptability of the intervention will be measured through “Yes” or “No” questions on usefulness, appropriateness, sharing, learning and support in diabetes management and behavior change [52].

### Physical measurements

Blood pressure, height and weight will be measured following standard guidelines from the WHO STEPS Survey, Nepal [53].

Height: Height will be measured using a portable stadiometer and values will be recorded in centimetres. We will ensure that the participants remove footwear or any accessories on the head and stand on a flat surface with their feet 10 cm apart heels against the wall, and knees straight.

Weight: Weight is measured using a portable digital Seca weighing scale, and values will be recorded in kilograms. We will ensure that the participant is wearing light clothing and does not wear footwear.

Blood pressure: We will use an Omron digital automatic blood pressure monitor with universal-sized cuffs to measure blood pressure. After asking the participant to sit and rest for 15 minutes, we will take three readings of blood pressure, with an interval of 3 minutes each between each reading.

### Haemoglobin A1c (HbA1c) measurement

The primary outcome measure is change in HbA1c level (blood test) expressed as a percentage (%) from baseline to follow up (at 6 months). Blood tests will be performed at the Department of Clinical Biochemistry, Dhulikhel Hospital. The results will be recorded in the participant’s record form for the study. Participant will not incur any cost for this test.

### Participant timeline

**Fig 3:**
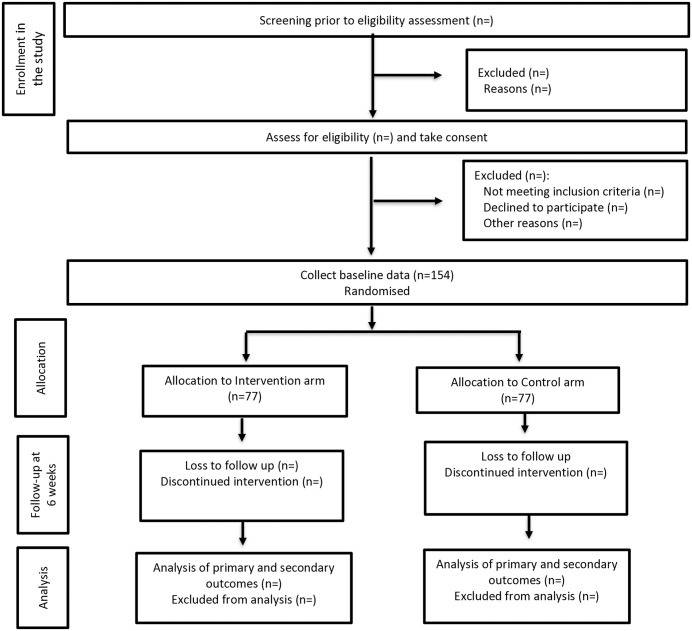
CONSORT 2010 flow diagram.

A CONSORT 2010 flow diagram [54] showing the participants timeline is displayed in Fig 3.

### Sample size

The sample size was calculated using Stata Version 13.0 for comparing two independent means [55]. Considering a mean difference (standard deviation) in HbA1c to be 0.86 (±1.08) in the intervention group and 0.18 (±1.53) in the control group from baseline to after 6 months’ of intervention [56] a sample size of 122 was calculated initially to achieve a power of 80% at 5% level of significance. Adjusting to a 20% attrition rate, the sample size was increased to 153. To consider an equal number of participants in each group, we plan to include 154 participants, with 77 participants in each arm.

### Randomisation, sequence generation and allocation concealment

A simple randomization technique [57] will be used to assign participants to either intervention or control group on 1:1 basis. Concealment of allocation will be done using sequentially numbered, opaque, sealed envelope (SNOSE) technique [58]. An open-source random number sequence generator (random.org) will be used to generate random numbers without duplication in two columns (77 each) from 1 to 154 indicating column one for intervention group and column two for control group [59]. The generated numbers in column one and column two will be kept in sequential order in two separate opaque sealed envelopes with a number labelled on outside. The lead researcher will generate the random number and prepare the envelopes while the research assistant will assign the participants to intervention or control group accordingly. Due to nature of the intervention, blinding of participants is not feasible as the participants will be aware of whether they are receiving the text-based mHealth intervention or just usual care.

### Data collection procedure

#### Baseline data collection.

Baseline data collection will be collected using a structured questionnaire inbuilt within KoboToolbox, an open source digital data collection platform using KoboCollect application [60] in android tablets. This system allows for real-time, accurate and secure data capture helping to reduce data entry errors during data entry and ensuring completeness and consistency. Access to the data will be restricted to authorised persons through password protection and user-specific permissions to maintain confidentiality and security. Details of measurements are mentioned in Table 1. The data collection tool will include questions on: A. Socio-demographic factors: Age, sex, ethnicity, religion, marital status, education and occupation and B. Family history, individual co-morbid condition and complications of diabetes

#### Post intervention/follow up data collection.

Follow-up data collection will be conducted after six months after the baseline assessment using the same set of repeated measures. In addition, the follow up questionnaire will include structured questions designed to assess the acceptability of the text message intervention.

#### Retention of the study participants.

To enhance participant retention and minimise loss to follow up, several strategies will be implemented. Firstly, participants will be clearly informed about the study objectives, procedure and importance of continued participation during the informed consent process. Ongoing communication will be maintained through phone calls during the intervention period. For the follow up, a minimum of three phone calls will be made to reach participant. If contact cannot be established after three attempts, the participant will be recorded as loss to follow up.

### Data management

The demographic and baseline characteristics will be reported using descriptive statistics such as frequencies and percentages for categorical variables, mean and standard deviation or median, quartiles and IQR for continuous variables.

The primary outcome is the change in hemoglobin A1c from baseline to six months. Given the repeated measurements and the two-group design (intervention vs. control), a two-way repeated measures ANOVA will be used to assess the main effects of time, intervention group, and the time × intervention interaction. This approach accounts for the correlation between repeated measures within participants and allows simultaneous assessment of both between-group and within-group changes. Estimated mean differences will be reported with 95% confidence intervals, and statistical significance will be set at p < 0.05. If the assumptions of ANOVA (e.g., normality, sphericity) are not met, appropriate adjustments such as the Greenhouse-Geisser correction or linear mixed-effects models will be considered.

Secondary outcomes, including self-efficacy, perceived support, diabetes-related stress, health-related quality of life, and self-care activities, will be summarized descriptively using mean ± SD or median (IQR), as appropriate. Between-group comparisons for these subjective outcomes will be conducted using non-parametric tests such as the Mann-Whitney U test for independent samples or the Wilcoxon signed-rank test for within-group changes. Categorical secondary outcomes will be analysed using chi-square or Fisher’s exact tests, as appropriate.

All analysis will be carried out based on the intention -to -treat principle. Any missing data will be reported and if needed will be addressed using the multiple imputation method.

### Monitoring

An independent Data Safety and Monitoring Committee (DSMC) has been established, consisting of five members: an endocrinologist, a medical technologist, a statistician, a health economist, and an ethics expert. The committee is responsible for overseeing participant safety, ensuring data quality, and maintaining the overall integrity of the study.

The study is registered in a recognised clinical trial registry and will be conducted in accordance with the approved protocol. Any deviations from the protocol will be documented and reported accordingly.

The study team will regularly monitor participant recruitment, intervention adherence, and data completeness throughout the trial. Given the non-clinical nature of the intervention, no adverse effects are anticipated. However, any adverse events that do occur will be appropriately recorded and reported.

Access to the final, de-identified dataset will be restricted to the study team to ensure data confidentiality and security.

### Patient and public involvement

Participants, including healthcare workers, will be involved in the development of the intervention through formative research. Their insights and experiences will help shape the content and delivery of the mobile health intervention. Once the initial version of the intervention is developed, the intervention will be pre-tested among similar participants to gather feedback on its relevance, usability, and acceptability. Based on their input, the intervention content will be refined to better align with the needs and preferences of the target population.

### Ethics and dissemination

Ethical approval was sought from the School Research Ethics and Integrity Committee at the University of Huddersfield, United Kingdom, the Ethical Review Board (ERB) of the Nepal Health Research Council and Institutional Review Committee (IRC), Dhulikhel Hospital, Nepal. The study will adhere to the ethical principles outlined in the National Ethical Guidelines for Human Research in Nepal 2022 [61].

Written informed consent will be sought from the participants during different stages of the study. The protocol for the trial has been developed and registered in the clinical trial registry (clinicalTrials.gov). The study will be conducted in accordance with the protocol, and any changes will be notified.

Participant confidentiality will be maintained in accordance with the general data requirements of the General Data Protection Regulation (GDPR) and Data Protection Act 2018, the University of Huddersfield, United Kingdom. Participants will be assigned unique identifiers or codes to ensure anonymity. No personal identifiers will be revealed beyond the researcher(s) managing the data, and it will not be used while reporting the results or sharing data.

The result of this study will be published in peer-reviewed scientific journals and presented at scientific conferences. In addition, the final research report will be made publicly available in a digital repository, University of Huddersfield, United Kingdom.

### Trial status

Formative research to inform the development of the intervention has been completed. Recruitment of participants for this study has not commenced yet. Participant recruitment is expected to begin in August 2025 and conclude by September 2025. Baseline data collection is anticipated to take place between October and November 2025. Follow-up data collection will occur six months after the baseline during May to June 2026. Baseline results are expected to be available by end of December 2025 with follow-up results anticipated by July 2026.

## Discussion

The rising burden of diabetes and the sub-optimal capacity of public health facilities to deal with the increasing burden of the disease in Nepal calls for an alternative, innovative and cost effective approach to reach and ensure continuity of care for people with Type 2 diabetes.

Self-management as an approach to disease management has been proven to be cost-effective with a progressive effect on clinical, psychological and behavioural aspects of diabetes as well as quality of life [15]. mHealth, as a digital transformation, is increasingly used to manage chronic diseases [17]. An overview of systematic reviews suggested mHealth intervention as an effective approach for self-management of Type 2 diabetes with a clinically meaningful improvement in glycemic control [20]. It can be a cost-effective way to deliver care and improve health outcomes in resource limited setting like Nepal [28].

Despite the availability of a body of evidence supporting mHealth as an effective approach to improving self-management among people with Type 2 diabetes, its application is yet to be evaluated in Nepal. This study thus aims to evaluate and determine the acceptability of a text-based mHealth intervention that supports self-management in people with Type 2 diabetes in Nepal.

The intervention is comprehensive in terms of engagement of users and service providers in its development process guided by a behaviour change theory. If proven to be effective, it can be integrated into routine care for people with Type 2 diabetes to improve self-management of their condition.

## Supporting information

S1 File SPIRIT checklist. (DOCX)

S2 File PLOS’ questionnaire on inclusivity in global research. (DOCX)

S3 File Study protocol. (DOCX)

## References

[1] Diabetes. Accessed 2022 March 30 https://www.who.int/news-room/fact-sheets/detail/diabetes

[2] International Diabetes Federation. Diabetes atlas. International Diabetes Federation; 2025.

[3] Dhungana RR, Pandey AR, Shrestha N. Trends in the prevalence, awareness, treatment, and control of hypertension in Nepal between 2000 and 2025: a systematic review and meta-analysis. Int J Hypertens. 2021;2021:6610649. doi: 10.1155/2021/6610649 33747559 PMC7952181

[4] Shrestha DB, Budhathoki P, Sedhai YR, Marahatta A, Lamichhane S, Nepal S, et al. Type 2 diabetes mellitus in nepal from 2000 to 2020: a systematic review and meta-analysis. F1000Research. 2021;10. 10.12688/f1000research.53970.1 PMC8459622 34621512

[5] Institute for Health Metrics and Evaluation IHME. GBD compare data visualization. 2025.

[6] International Diabetes Federation. Nepal diabetes country report 2011 — 2050. 2025.

[7] Upreti SR, Lohani GR, Magtymova A, Dixit LP. Strengthening policy and governance to address the growing burden of diabetes in Nepal. WHO South East Asia J Public Health. 2016;5(1):40–3. doi: 10.4103/2224-3151.206551 28604396

[8] Dhimal M, Bista B, Bhattarai S, Dixit LP, Hyder MKA, Agrawal N. Report of non communicable disease risk factors: STEPS survey Nepal 2019. Kathmandu, Nepal. 2020.

[9] Gyawali B, Ferrario A, van Teijlingen E, Kallestrup P. Challenges in diabetes mellitus type 2 management in Nepal: a literature review. Glob Health Action. 2016;9:31704. doi: 10.3402/gha.v9.31704 27760677 PMC5071649

[10] Huda MD, Rahman M, Rahman MM, Islam MJ, Haque SE, Mostofa MG. Readiness of health facilities and determinants to manage diabetes mellitus: evidence from the nationwide service provision assessment survey of Afghanistan, Bangladesh and Nepal. BMJ Open. 2021;11(12):e054031. doi: 10.1136/bmjopen-2021-054031

[11] Ghimire U, Shrestha N, Adhikari B, Mehata S, Pokharel Y, Mishra SR. Health system’s readiness to provide cardiovascular, diabetes and chronic respiratory disease related services in Nepal: analysis using 2015 health facility survey. BMC Public Health. 2020;20(1):1163. doi: 10.1186/s12889-020-09279-z 32711487 PMC7382840

[12] Adhikari B, Pandey AR, Lamichhane B, Kc SP, Joshi D, Regmi S. Non-communicable disease service readiness in Nepal: a further analysis of Nepal health facility survey-2021. medRxiv. 2023. doi: 10.1101/2023.02.07.23285512 PMC10335515 37423630

[13] American Association of Diabetes Educators. An effective model of diabetes care and education: revising the AADE7 self-care behaviors®. Diabetes Educ. 2020;46(2):139–60. doi: 10.1177/0145721719894903 31928334

[14] Powers MA, Bardsley J, Cypress M, Duker P, Funnell MM, Fischl AH, et al. Diabetes self-management education and support in type 2 diabetes: a joint position statement of the American diabetes association, the American association of diabetes educators, and the academy of nutrition and dietetics. Diabetes Edu. 2017;43(1):40–53. 10.1016/j.jand.2015.05.012 26054423

[15] Panagioti M, Richardson G, Small N, Murray E, Rogers A, Kennedy A, et al. Self-management support interventions to reduce health care utilisation without compromising outcomes: a systematic review and meta-analysis. BMC Health Serv Res. 2014;14:356. doi: 10.1186/1472-6963-14-356 25164529 PMC4177163

[16] Sukartini T, Nursalam N, Pradipta RO, Ubudiyah M. Potential methods to improve self-management in those with type 2 diabetes: a narrative review. Int J Endocrinol Metab. 2023;21(1):e119698. doi: 10.5812/ijem-119698 37038539 PMC10082325

[17] Kang H, Park H-A. A mobile app for hypertension management based on clinical practice guidelines: development and deployment. JMIR Mhealth Uhealth. 2016;4(1):e12. doi: 10.2196/mhealth.4966 26839283 PMC4756253

[18] Fadilah SZ, Susanti IA, Setyorini DY, Pradipta RO. Effectiveness of mobile-based health interventions for the management of hypertensive patients: a systematic review. J Ners. 2020;15(1Sp):238–45. doi: 10.20473/jn.v15i1sp.19022

[19] Johnston L, Zemanek J, Reeve MJ, Grills N. The evidence for using mHealth technologies for diabetes management in low- and middle-income countries. J Hosp Manag Health Policy. 2018;2:35–35. doi: 10.21037/jhmhp.2018.07.01

[20] Kitsiou S, Paré G, Jaana M, Gerber B. Effectiveness of mHealth interventions for patients with diabetes: an overview of systematic reviews. PLoS One. 2017;12(3):e0173160. doi: 10.1371/journal.pone.0173160 28249025 PMC5332111

[21] Mao Y, Lin W, Weng J, Chen G. The clinical outcomes and effectiveness of mHealth interventions for diabetes and hypertension: a systematic review and meta-analysis. medRxiv. 2020.

[22] Lu X, Yang H, Xia X, Lu X, Lin J, Liu F, et al. Interactive mobile health intervention and blood pressure management in adults. Hypertension. 2019;74(3):697–704. doi: 10.1161/HYPERTENSIONAHA.119.13273 31327259

[23] Wang Y, Min J, Khuri J, Xue H, Xie B, A Kaminsky L, et al. Effectiveness of mobile health interventions on diabetes and obesity treatment and management: systematic review of systematic reviews. JMIR Mhealth Uhealth. 2020;8(4):e15400. doi: 10.2196/15400 32343253 PMC7218595

[24] Xiong S, Berkhouse H, Schooler M, Pu W, Sun A, Gong E, et al. Effectiveness of mHealth interventions in improving medication adherence among people with hypertension: a systematic review. Curr Hypertens Rep. 2018;20(10):86. doi: 10.1007/s11906-018-0886-7 30088110

[25] CBS. National population and housing census 2021. 2021.

[26] Bhandari B, Narasimhan P, Jayasuriya R, Vaidya A, Schutte AE. Effectiveness and acceptability of a mobile phone text messaging intervention to improve blood pressure control (TEXT4BP) among patients with hypertension in Nepal: A Feasibility Randomised Controlled Trial. Glob Heart. 2022;17(1):13. doi: 10.5334/gh.1103 35342691 PMC8877709

[27] Ni Z, Atluri N, Shaw RJ, Tan J, Khan K, Merk H, et al. Evaluating the feasibility and acceptability of a mobile health-based female community health volunteer program for hypertension control in rural Nepal: cross-sectional study. JMIR Mhealth Uhealth. 2020;8(3):e15419. doi: 10.2196/15419 32149712 PMC7091025

[28] Tuitui RL, Bhatt A, Pradhan S, Hutchinson G, Gowland S, Saha S, et al. Using mobile health to strengthen the communication skills for effective delivery of health information in Nepal: a qualitative study of the perspectives of female community health volunteers. J Glob Health Econ Pol. 2022;2. doi: 10.52872/001c.36187

[29] Chan A-W, Tetzlaff JM, Altman DG, Laupacis A, Gøtzsche PC, Krleža-Jerić K, et al. SPIRIT 2013 statement: defining standard protocol items for clinical trials. Ann Intern Med. 2013;158(3):200–7. doi: 10.7326/0003-4819-158-3-201302050-00583 23295957 PMC5114123

[30] Eysenbach G, CONSORT-EHEALTH Group. CONSORT-EHEALTH: improving and standardizing evaluation reports of Web-based and mobile health interventions. J Med Internet Res. 2011;13(4):e126. doi: 10.2196/jmir.1923 22209829 PMC3278112

[31] Dhulikhel H. About dhulikhel hospital. Accessed 2025 April 21 https://dhulikhelhospital.org/about-dh/

[32] Excellence NIFC. Behaviour change: individual approaches (NICE Guidelines PH49). National Institute for Health and Care Excellence; 2014. https://www.nice.org.uk/guidance/ph49

[33] Skivington K, Matthews L, Simpson SA, Craig P, Baird J, Blazeby JM, et al. A new framework for developing and evaluating complex interventions: update of medical research council guidance. BMJ. 2021;374. 10.1136/bmj.n2061 PMC8482308 34593508

[34] Davis R, Campbell R, Hildon Z, Hobbs L, Michie S. Theories of behaviour and behaviour change across the social and behavioural sciences: a scoping review. Health Psychol Rev. 2015;9(3):323–44. doi: 10.1080/17437199.2014.941722 25104107 PMC4566873

[35] Michie S, van Stralen MM, West R. The behaviour change wheel: a new method for characterising and designing behaviour change interventions. Implement Sci. 2011;6:42. doi: 10.1186/1748-5908-6-42 21513547 PMC3096582

[36] Yusoff MSB. ABC of content validation and content validity index calculation. EIMJ. 2019;11(2):49–54. doi: 10.21315/eimj2019.11.2.6

[37] Chai LK, May C, Collins CE, Burrows TL. Development of text messages targeting healthy eating for children in the context of parenting partnerships. Nutr Diet. 2019;76(5):515–20. doi: 10.1111/1747-0080.12498 30426627

[38] Ritter PL, Lorig K, Laurent DD. Characteristics of the Spanish- and English-language self-efficacy to manage diabetes scales. Diabetes Educ. 2016;42(2):167–77. doi: 10.1177/0145721716628648 26846336

[39] Kerari A. The psychometric properties of the diabetes self-efficacy scale in saudis with type 2 diabetes. Nurs Open. 2023;10(9):6408–15. doi: 10.1002/nop2.1890 37319293 PMC10416040

[40] Mankan T, Erci B, Bahçecioğlu Turan G, Aktürk Ü. Turkish validity and reliability of the diabetes self-efficacy scale. Int J Nurs Sci. 2017;4(3):239–43. doi: 10.1016/j.ijnss.2017.05.001 31406747 PMC6626172

[41] Dwa N, Panthee B. Perceived self-efficacy and self-care practices among diabetic patients in a Tertiary Hospital, Nepal. J Dia Endo Assoc Nepal. 2021;5(1):25–32. doi: 10.3126/jdean.v5i1.38801

[42] Zimet GD, Dahlem NW, Zimet SG, Farley GK. The multidimensional scale of perceived social support. J Personal Assess. 1988;52(1):30–41. doi: 10.1207/s15327752jpa5201_2

[43] Tonsing K, Zimet GD, Tse S. Assessing social support among South Asians: the multidimensional scale of perceived social support. Asian J Psychiatr. 2012;5(2):164–8. doi: 10.1016/j.ajp.2012.02.012 22813661

[44] Akter J, Islam RM, Chowdhury HA, Selim S, Biswas A, Mozumder TA, et al. Psychometric validation of diabetes distress scale in Bangladeshi population. Sci Rep. 2022;12(1):562. doi: 10.1038/s41598-021-04671-0 35022493 PMC8755848

[45] WHOQOL User Manual. 1998. https://iris.who.int/bitstream/handle/10665/77932/WHO_HIS_HSI_Rev.2012.03_eng.pdf?sequence=1

[46] Almarabheh A, Salah AB, Alghamdi M, Al Saleh A, Elbarbary A, Al Qashar A, et al. Validity and reliability of the WHOQOL-BREF in the measurement of the quality of life of Sickle disease patients in Bahrain. Front Psychol. 2023;14:1219576. doi: 10.3389/fpsyg.2023.1219576 37720642 PMC10503438

[47] Kalfoss MH, Reidunsdatter RJ, Klöckner CA, Nilsen M. Validation of the WHOQOL-Bref: psychometric properties and normative data for the Norwegian general population. Health Qual Life Outcomes. 2021;19(1):13. doi: 10.1186/s12955-020-01656-x 33413455 PMC7792093

[48] Sreedevi A, Cherkil S, Kuttikattu DS, Kamalamma L, Oldenburg B. Validation of WHOQOL-BREF in Malayalam and determinants of quality of life among people with type 2 diabetes in Kerala, India. Asia Pac J Public Health. 2016;28(1 Suppl):62S-69S. doi: 10.1177/1010539515605888 26419636 PMC4803787

[49] Joshi KD, Thapa J, Bhandary S. Assessing quality of life among older persons using WHOQOL-BREF tool – a pilot study in Chandragiri, Municipality. J Gen Pract Emerg Med Nepal. 2023;10(15):18–23. doi: 10.59284/jgpeman232

[50] Toobert DJ, Hampson SE, Glasgow RE. The summary of diabetes self-care activities measure: results from 7 studies and a revised scale. Diabetes Care. 2000;23(7):943–50. doi: 10.2337/diacare.23.7.943 10895844

[51] Jalaludin M, Fuziah M, Hong J, Mohamad Adam B, Jamaiyah H. Reliability and validity of the revised summary of diabetes self-care activities (SDSCA) for Malaysian children and adolescents. Malays Fam Physician. 2012;7(2–3):10–20. 25606251 PMC4170432

[52] Dobson R, Whittaker R, Jiang Y, Shepherd M, Maddison R, Carter K, et al. Text message-based diabetes self-management support (SMS4BG): study protocol for a randomised controlled trial. Trials. 2016;17:179. doi: 10.1186/s13063-016-1305-5 27039300 PMC4818933

[53] Dhimal M, Bista B, Bhattarai S, Dixit LP, Hyder MKA, Agrawal N, et al. Report of non communicable disease risk factors: STEPS survey Nepal 2019. Nepal Health Research Council; 2019.

[54] Schulz KF, Altman DG, Moher D. CONSORT 2010 statement: updated guidelines for reporting parallel group randomised trials. J Pharmacol Pharmacother. 2010;1(2):100–7. doi: 10.4103/0976-500X.72352 21350618 PMC3043330

[55] StataCorp. Stata: release 13. Statistical software. College Station, TX: StataCorp LP; 2013.

[56] Shariful Islam SM, Niessen LW, Ferrari U, Ali L, Seissler J, Lechner A. Effects of mobile phone SMS to improve glycemic control among patients with type 2 diabetes in Bangladesh: a prospective, parallel-group, randomized controlled trial. Diabetes Care. 2015;38(8):e112-3. doi: 10.2337/dc15-0505 26207059

[57] Kang M, Ragan BG, Park J-H. Issues in outcomes research: an overview of randomization techniques for clinical trials. J Athl Train. 2008;43(2):215–21. doi: 10.4085/1062-6050-43.2.215 18345348 PMC2267325

[58] Sil A, Kumar P, Kumar R, Das NK. Selection of control, randomization, blinding, and allocation concealment. Indian Dermatol Online J. 2019;10(5):601–5. doi: 10.4103/idoj.IDOJ_149_19 31544090 PMC6743387

[59] Random sequence generator. Accessed 2023 October 1 https://www.random.org/sequences/

[60] Harvard Humanitarian Initiative. KoBoToolbox. 2019.

[61] NHRC. National ethical guidelines for health research in Nepal. Kathmandu, Nepal: Nepal Health Research Council. 2022.

