# Supplementary material for: Self-management of type 2 diabetes using a co-designed text based mobile health (mHealth) intervention in Nepal: A study protocol for randomised controlled trial
Source: PLoS One. 2025 Nov 4;20(11):e0335333. doi: 10.1371/journal.pone.0335333 (PMC12585027; doi:10.1371/journal.pone.0335333)
Supplement: S3 File — (DOCX) [file pone.0335333.s003.docx]

#### Title of the study:

Self-management of type 2 diabetes using a co-designed text based mobile health (mhealth) intervention in Nepal: a randomized controlled trial

#### Summary of the proposed research protocol (within 200 words)

The overall burden of diabetes is rising, especially in low and middle-income countries such as Nepal. In Nepal, access to diabetes care varies by socio-economic and geographical factors, posing significant challenges to prevention and control. Self-management plays a crucial role in the prevention and control of diabetes. Mobile health (mHealth) technology is increasingly popular for managing chronic diseases, demonstrating its feasibility, acceptability, and effectiveness in enhancing health outcomes and encouraging behavior change. However, its impact on improving self-management among people with type 2 diabetes in Nepal remains unexplored. This randomised controlled trial aims to evaluate a 6-month text based mhealth intervention that support self- management among people with type 2 diabetes in Nepal. The primary outcome includes change in HbA1c at 6 months. Secondary outcomes include behaviour change for diabetes self-management activities, self-efficacy, perceived support, diabetes related stress, quality of life and acceptability of the intervention acceptability. In addition, an economic evaluation will be conducted using a patient perspective. Primary and secondary outcomes will be summarized descriptively, and comparisons will be made using chi-square tests for categorical data and independent sample t-tests for continuous data using SPSS. A cost-effectiveness analysis will be conducted using the incremental cost-effectiveness ratio (ICER).

#### DETAIL OF THE STUDY

**B.1. BACKGROUND:**

The number of people living with diabetes has increased globally and is on rapid rise among low and middle income countries (LMICs) ([WHO, 2021](#_ENREF_52)). In 2021, there were 537 million people living with diabetes and this number is expected to rise to 643 million by 2030 and 783 million by 2045 ([IDF, 2021](#_ENREF_21)). More than three in four adults with diabetes are living in LMICs. Diabetes accounted for 6.6 million deaths in 2021 ([IDF, 2021](#_ENREF_21)) and is a major cause of blindness, kidney failure, heart attack, stroke and lower limb amputation ([WHO, 2021](#_ENREF_52)). Following the global trend, the prevalence of diabetes has been increasing in Nepal ([Dhungana et al., 2021](#_ENREF_9); [Shrestha et al., 2021](#_ENREF_42)). The International Diabetes Federation (IDF) estimated a prevalence of 8.7% among Nepalese population and the proportion is to rise to 9.4% by 2045 ([International Diabetes Federation, 2021](#_ENREF_22)).

The burgeoning burden of disease may place serious challenges on the resource constrained national health system due to complications resulting from the disease, coupled with issues around service availability & readiness and limited awareness among the people living with diabetes ([Ghimire et al., 2020](#_ENREF_16); [Gyawali et al., 2016](#_ENREF_19)).

Access to care is of utmost importance for people with diabetes in the course of their disease however, the availability and utilisation of diabetes care services vary across socio-economic and geographical distribution indicating challenges in accessing the diabetes care services ([Upreti et al., 2016](#_ENREF_49)). WHO STEPS survey Nepal 2019 reported about three-fourth (73.5%) of the people were not aware of their raised blood sugar. Despite being aware of their condition, about 6% were not on treatment. Similarly, only 70% of adults with raised blood sugar reported compliance to medications to control their blood sugar ([Dhimal M et al., 2020](#_ENREF_8)). In addition, public health facilities in Nepal are not ready to deal with the increasing burden of NCDs including diabetes in terms of qualified health staff, training, availability of equipment and medicines as well as availability of diabetes services in general ([Adhikari et al., 2023](#_ENREF_4); [Ghimire et al., 2020](#_ENREF_16); [Huda et al., 2021](#_ENREF_20)).

Early diagnosis, treatment and self-management are essential for the prevention and control of the disease ([WHO, 2021](#_ENREF_52)). Self-management refers to the activities and behaviour undertaken by the individuals to control and treat their conditions to improve their health outcomes with support from family members, friends and relatives and in consultation with health service providers ([Powers et al., 2017](#_ENREF_39)). The American Association of Diabetes Educators (AADE) has identified seven self-care behaviours for successful and effective self-management of diabetes as healthy coping, healthy eating, being active, taking medication, monitoring, reducing risks and problem solving ([AADE, 2020](#_ENREF_1)). Self-management has been proved to be cost effective through reduction in hospital admissions and health care costs ([Panagioti et al., 2014](#_ENREF_38)). Besides, it is shown to improve HbA1c level and has a positive effect on other clinical, psychosocial, and behavioural aspects of diabetes along with improvement in quality of life by reducing onset and/or advancement of diabetes complications ([Powers et al., 2017](#_ENREF_39); [Sukartini et al., 2023](#_ENREF_46)).

With the development of technology, the application of mobile health (mhealth) is becoming increasingly popular in the self-care of chronic diseases ([Kang & Park, 2016](#_ENREF_25)). mhealth interventions have been proved to be a therapeutic strategy that could improve the diabetes management despite their economic status ([Fadilah et al., 2020](#_ENREF_12); [Johnston et al., 2018](#_ENREF_24); [Kitsiou et al., 2017](#_ENREF_26); [Lu et al., 2019](#_ENREF_28); [Mao et al., 2020](#_ENREF_30); [Wang et al., 2020](#_ENREF_50); [Xiong et al., 2018](#_ENREF_54)). According to the National Population and Housing Census report 2021 ([CBS, 2021](#_ENREF_6)), about three-fourth (73%) of the households have at least some form of smartphone penetration and 37.9% household with internet access in Nepal.

**STATEMENT OF THE PROBLEM/ RATIONALE/ NEED OF THE STUDY:**

In the resource limited countries like Nepal, mhealth technology can be a cost-effective approach to deliver care and improve the health outcomes. There are studies demonstrating the feasibility, acceptability ([Bhandari et al., 2022](#_ENREF_5); [Ni et al., 2020](#_ENREF_36)) and even effectiveness ([Bhandari et al., 2022](#_ENREF_5)) of mhealth in improving the health outcomes for hypertension in Nepal ([Bhandari et al., 2022](#_ENREF_5); [Ni et al., 2020](#_ENREF_36)). There is also existing evidence of mhealth as an effective means to communicate health related information and supporting positive behaviour change in a short period of time ([Tuitui et al., 2022](#_ENREF_48)). Despite availability of body of evidence on acceptability of mhealth in improving service utilization and health outcomes for chronic conditions, the application of behaviour change interventions through mhealth for self-management of diabetes is yet to be evaluated.

**OBJECTIVES OF THE RESEARCH:**

***General objective (s):***

This study aims to develop and evaluate a text based mhealth intervention that support self-management in people with Type 2 Diabetes using a co-designed approach

***Specific objective(s):***

1. To identify the barriers and facilitators in adoption of text based mhealth intervention in self-management of type 2 diabetes
2. To explore the perspective of service providers and end users on use of text based mhealth for self-management of type 2 diabetes
3. To develop a theory based contextual text based mhealth intervention that support self-management in people with type 2 diabetes
4. To determine the effectiveness, cost-effectiveness and acceptability of text based mhealth intervention in supporting self-management in people with type 2 diabetes

**STUDY SITE AND JUSTIFICATION:**

The study will be conducted in Dhulikhel hospital of Kavrepalanchowk district, Nepal. Dhulikhel hospital is a tertiary level hospital, catering comprehensive and quality health services to about 2.5 million people having diverse characteristics in terms of ethnicity, residence, socio-economic status and health conditions with particular focus on rural and unserved population.

**METHODS/ METHODOLOGY:**

***Mixed Research Design***

We will conduct a qualitative study to explore perspectives of service providers and users on use of text based mhealth to support self-management among people with type 2 diabetes together with identifying potential barriers and facilitators for implementation of the intervention. The study population will include people with type 2 diabetes aged 18 years and above. We will further include service providers involved in the delivering diabetes care services. We will collect qualitative data using In-Depth Interview (IDI), and Focused Group Discussion (FGD).

Further to this, the study will include a 6-month text-based mhealth intervention and employs a two-arm parallel group randomised control trial to evaluate the effectiveness, cost effectiveness and acceptability of a text-based mhealth intervention in improving self-management among people with type 2 diabetes.

***Study design:***

This study is a 6 month text-based mhealth intervention and employs a two-arm parallel group randomised control trial to evaluate the effectiveness and acceptability of a text-based mhealth intervention in improving self-management among people with type 2 diabetes in Dhulikhel Hospital, Kavrepalanchowk, Nepal.

***Study Variables***

1. Socio-demographic factors: Age, sex, ethnicity, religion, marital status, education and occupation
2. Health related characteristics: Family history, individual co-morbid condition and complications of diabetes
3. Self-efficacy: Diabetes Self Efficacy Scale (DSES) is an 8-item with a scale ranging from 1 (not at all confident) to 10 (totally confident). The score for the scale is the mean of eight items where higher number indicated higher self-efficacy ([Ritter et al., 2016](#_ENREF_44)). This scale is found to have good internal consistency and test-retest validity ([Kerari, 2023](#_ENREF_30); [Mankan et al., 2017](#_ENREF_34)) and have been modified and used in Nepalese context ([Dwa & Panthee, 2021](#_ENREF_12)).
4. Perceived support: Multidimensional Scale of Perceived Social Support is a 12-item, 7-point Likert scale (1 as very strongly disagree to 7 as very strongly agree) to assess perceived support from family, friends and significant other ([Zimet et al., 1988](#_ENREF_60)). Nepalese version of this scale is found to have good construct validity and reliability ([Tonsing et al., 2012](#_ENREF_51)).
5. Diabetes related stress: Diabetes Distress Scale consists of 17 questions in total: Emotional burden (5 questions), Physician related distress (4 questions), Regimen related distress (5 questions) and Interpersonal distress (3 questions). Responses for the question ranges from 1 (slight problem) to 6 (very serious problem). Mean is calculated for each subscale. This scale is found to be valid and reliable tool to assess distress among people with type 2 diabetes in context similar to Nepal ([Akter et al., 2022](#_ENREF_3)).
6. Quality of life: WHOQOL- BREF consists of 26 items across four domains: physical, psychological, social and environment including the questions on overall quality of life and general health. The score ranges from 1 to 5 on a response scale. Higher scores denote higher quality of life ([WHO, 1998](#_ENREF_56)). This tool is found have good internal consistency and validity in assessing the quality of life of people across different cultures ([Almarabheh et al., 2023](#_ENREF_4); [Kalfoss et al., 2021](#_ENREF_27); [Sreedevi et al., 2016](#_ENREF_49)) including Nepal ([Joshi et al., 2023](#_ENREF_26)).We will use Nepali version of WHOQOL-BREF.
7. Self-care activities: Revised scale of Summary of Diabetes Self Care Activities measure consists of core set of 11 items from Summary of Diabetes Self Care Activities measure and additional 14 questions ([Toobert et al., 2000](#_ENREF_52)). It is found to be valid and reliable tool for assessing self-care behavior among adults with diabetes ([Jalaludin et al., 2012](#_ENREF_24)).
8. Economic evaluation: Economic evaluation of the intervention will be done using the cost information obtained at baseline and follow up (after 6 month) including the cost of development and delivery of mhealth intervention and Quality-adjusted Life Years (QALYs).
9. Acceptability of intervention: Acceptability of the intervention will be measured through “Yes” or “No” questions on usefulness, appropriateness, sharing, learning and support in diabetes management and behavior change ([Dobson et al., 2016](#_ENREF_11)).

***Expected outcomes of the research***

This study aims to evaluate and determine acceptability of a text based mhealth intervention that support self-management in people with type 2 diabetes in Nepal. If proven to be effective, it can be integrated into routine care for people with type 2 diabetes to improve self-management for their condition.

**STUDY POPULATION AND SAMPLE**

***Participants/ study population:***

The study population include people with type 2 diabetes visiting the diabetic clinic at Dhulikhel hospital and service providers who are directly involved in providing care for people with type 2 diabetes

***Selection criteria:***

***Inclusion criteria:***

Inclusion criteria for the study include a. adults aged 18 years and above b. clinical diagnosis of type 2 diabetes c. participant who have a mobile phone and is able to read the message on their own or with the help of family members.

***Exclusion criteria:***

Participants are not eligible if they are a. not physically or mentally able, not able to provide consent b. pregnant in case of female.

***Sampling method/ technique:***

Each person visiting the diabetic clinic in Dhulikhel hospital will be screened for eligibility. Those meeting the eligibility criteria will be approached to participate in the study. The participants will be recruited consecutively until the target sample size is reached.

***Sampling Unit:***

Individual

***Sample size determination:***

**Qualitative:** The number of study participants for IDI will depend on representation of diverse individuals particularly age and gender; and theory of saturation. The availability of study participants will determine the number of FGDs and may range from 2 to 4 with 6 to 8 participants in each FGD.

**Quantitative:** Considering a mean difference (standard deviation) in HbA1c to be 0.86 (±1.08) in the intervention group and 0.18 (±1.53) in the control group from baseline to after 6 months’ intervention ([Islam et al., 2015](#_ENREF_23)) a sample size of 122 was calculated initially to achieve a power of 80% at 5% level of significance. Adjusting to 20% attrition rate, the sample size was increased to 153. To consider equal number of participants in each group, we plan to include a total of 154 participants with 77 participants in each arm.

***Data Collection Procedure:***

Formative research: We will identify potential participants for formative research which includes people with type 2 diabetes and health service providers in coordination with the team from Dhulikhel hospital. The participants will be approached by the lead researcher and inform about the objective of the study through the information sheet. Those who provide written consent to participate in the study will be recruited and involved in the study for interview and discussion.

Screening of participants for RCT: We will screen each person visiting the diabetic clinic in Dhulikhel hospital for the eligibility. Those meeting the eligibility criteria will be approached to participate in the study.

Written Informed consent for RCT: Those who meet the inclusion criteria and agree to participate in the study will be provided with the information sheet that contains information about the objective of the study, procedures, duration, potential risk and benefits, confidentiality, their voluntary participation and frequency of participation in the study. The participants will be provided an opportunity to ask questions and clarifications on anything they are not sure about and presented with a consent form to review and sign if they agree to participate.

A simple randomization technique ([Kang et al., 2008](#_ENREF_29)) will be used to assign participants to either intervention or control group on 1:1 basis. Concealment of allocation will be done using Sequentially numbered, opaque, sealed envelope (SNOSE) technique ([Sil et al., 2019](#_ENREF_46)).An open-source random number sequence generator (random.org) will be used to generate random numbers in two columns (77 each) from 1 to 154 indicating column one for intervention group and column two for control group ([random.org](#_ENREF_41)). The generated numbers in column one and column two will be kept in sequential order in two separate opaque sealed envelopes with a number labelled on outside. The lead researcher will generate the random number and prepare the envelopes while the research assistant will assign the participants to intervention or control group accordingly. A face to face interview will be conducted with people with type 2 diabetes using a structured questionnaire which includes questions related to self-efficacy, perceived support, stress, quality of life, self-care behavior and cost (disease related and development and delivery of intervention) including questions on acceptability of the intervention at follow up. Data will be collected at baseline and 6 months after the intervention.

***Data Collection Tools/ Measures:***

Diabetes Self Efficacy Scale (DSES) is an 8-item with a scale ranging from 1 (not at all confident) to 10 (totally confident). The score for the scale is the mean of eight items where higher number indicated higher self-efficacy (Ritter et al., 2016). This scale is found to have good internal consistency and test- retest validity (Kerari, 2023; Mankan et al., 2017) and have been modified and used in Nepalese context (Dwa & Panthee, 2021).

Multidimensional Scale of Perceived Social Support is a 12-item, 7-point Likert scale (1 as very strongly disagree to 7 as very strongly agree) to assess perceived support from family,friends and significant other (Zimet et al., 1988). Nepalese version of this scale is found to have good construct validity and reliability (Tonsing et al., 2012).

Diabetes Distress Scale consists of 17 questions in total: Emotional burden (5 questions), Physician related distress (4 questions), Regimen related distress (5 questions) and Interpersonal distress (3 questions). Responses for the question ranges from 1 (slight problem) to 6 (very serious problem). Mean is calculated for each subscale. This scale is found to be valid and reliable tool to assess distress among people with type 2 diabetes in context similar to Nepal (Akter et al., 2022).

WHOQOL- BREF consists of 26 items across four domains: physical, psychological, social and environment including the questions on overall quality of life and general health. The score ranges from 1 to 5 on a response scale. Higher scores denote higher quality of life (WHO, 1998). This tool is found have good internal consistency and validity in assessing the quality of life of people across different cultures (Almarabheh et al., 2023; Kalfoss et al., 2021; Sreedevi et al., 2016) including Nepal (Joshi et al., 2023).We will use Nepali version of WHOQOL-BREF.

Revised scale of Summary of Diabetes Self Care Activities measure consists of core set of 11 items from Summary of Diabetes Self Care Activities measure and additional 14 questions (Toobert et al., 2000). It is found to be valid and reliable tool for assessing self- care behavior among adults with diabetes (Jalaludin et al., 2012).

Economic evaluation: An economic evaluation will be done of the intervention considering the following aspects: a. Cost (Resource Measurement) The study will measure both direct and indirect costs, including medical expenses (e.g., medication, hospital charges) and non-medical costs (e.g., travel, lost income). These costs will be valued based on actual patient payments and market rates, excluding non-monetized factors like leisure time. b. Cost of the mHealth Intervention Costs associated with the intervention will be calculated using project data, converting monetary values from Nepalese currency to Great Britain Pounds (GBP) at the appropriate exchange rate. Unit costs will be based on actual records. c. User’s Perspective Cost Measurement Out-of-pocket expenses, including medical and non-medical costs, will be collected from participants retrospectively at baseline and prospectively during the study. Participants will record expenses in forms and diaries, which will be used during follow-up data collection. d. Effects Measurement (Health-related Outcomes) The study will assess health outcomes such as HbA1c levels, self-care management, and quality of life using the EuroQol-5 Dimension (EQ-5D) tool. This will help measure Quality-Adjusted Life Years (QALYs), using the Indian EQ-5D value set due to the cultural similarities between Nepal and India. e. Incremental Cost-Effectiveness Ratio (ICER) ICER will measure the economic value of the intervention by comparing the incremental costs and health outcomes (e.g., QALYs gained, HbA1c reduction) between the intervention and control groups.

Acceptability of the intervention will be measured through “Yes” or “No” questions on usefulness, appropriateness, sharing, learning and support in diabetes management and behavior change (Dobson et al., 2016).

Physical measurements

Blood pressure, height and weight will be measured following standard guideline for measurement from WHO STEPS Survey, Nepal (Dhimal M et al., 2019).

Height: Height will be measured using a portable stature meter and values will be recorded in centimetres. We will ensure that the participants removed footwear or any accessories on the head and stands on a flat surface with their feet 10 cm apart and heels against the wall with knees straight.

Weight: Weight is measured using a portable digital seca weighing scale and values will be recorded in kilograms. We will ensure that the participant is on light clothing and without footwear.

Blood pressure: We will use an Omron digital automatic blood pressure monitor with universal sized cuffs to measure blood pressure. Three readings of blood pressure will be taken with an interval of 3 minutes each between the readings after having asked the participant to sit and rest for 15 minutes.

Blood collection There are no specific conditions (fasting, special diets) required for patient. A trained lab person will collect the blood sample using aseptic technique. Laboratory technician and laboratory technologist are responsible for conducting this test and the test will be performed in a laboratory of Dhulikhel hospital. HbA1c test will be performed following standard protocols of the laboratory.

***Pretesting***

A pretesting of the questionnaire will be done among people with type 2 diabetes. In addition, the content developed will be pre-tested among participants similar to target group to improve on content validity.

***Validity and Reliability of tool***

Diabetes Self Efficacy Scale (DSES) is an 8-item with a scale ranging from 1 (not at all confident) to 10 (totally confident). The score for the scale is the mean of eight items where higher number indicated higher self-efficacy (Ritter et al., 2016). This scale is found to have good internal consistency and test- retest validity (Kerari, 2023; Mankan et al., 2017) and have been modified and used in Nepalese context (Dwa & Panthee, 2021). Multidimensional Scale of Perceived Social Support is a 12-item, 7-point Likert scale (1 as very strongly disagree to 7 as very strongly agree) to assess perceived support from family, friends and significant other (Zimet et al., 1988). Nepalese version of this scale is found to have good construct validity and reliability (Tonsing et al., 2012). Diabetes Distress Scale consists of 17 questions in total: Emotional burden (5 questions), Physician related distress (4 questions), Regimen related distress (5 questions) and Interpersonal distress (3 questions). Responses for the question ranges from 1 (slight problem) to 6 (very serious problem). Mean is calculated for each subscale. This scale is found to be valid and reliable tool to assess distress among people with type 2 diabetes in context similar to Nepal (Akter et al., 2022). WHOQOL- BREF consists of 26 items across four domains: physical, psychological, social and environment including the questions on overall quality of life and general health. The score ranges from 1 to 5 on a response scale. Higher scores denote higher quality of life (WHO, 1998). This tool is found have good internal consistency and validity in assessing the quality of life of people across different cultures (Almarabheh et al., 2023; Kalfoss et al., 2021; Sreedevi et al., 2016) including Nepal (Joshi et al., 2023). We will use Nepali version of WHOQOL-BREF. Revised scale of Summary of Diabetes Self Care Activities measure consists of core set of 11 items from Summary of Diabetes Self Care Activities measure and additional 14 questions (Toobert et al., 2000). It is found to be valid and reliable tool for assessing self- care behavior among adults with diabetes (Jalaludin et al., 2012). Blood pressure, height and weight will be measured following standard guideline for measurement from WHO STEPS Survey, Nepal (Dhimal M et al., 2019). HbA1c test will be performed in a laboratory affiliated to National Public Health Laboratory, Nepal. HbA1c test will be performed following standard protocols of the assigned laboratory.

Furthermore, to determine the validity of the developed intervention (text message), expert rating on the content will be used where experts (5-6) will be asked to rate each message on a scale of 1 to 4 where, 1=not relevant, 2=somewhat relevant, 3=quite relevant and 4=highly relevant. A content validity index will be calculated.

***Plan for specimen/data management:***

Participants will be assigned unique identifiers or codes to ensure anonymity. No personal identifiers will be revealed beyond the researcher(s) managing the data and it will not be used while reporting the results or sharing data. Confidentiality of the data will be protected in accordance with the requirements of the General Data Protection Regulation (GDPR) and Data Protection Act 2018, University of Huddersfield. Data will be kept confidentially in accordance with the general data requirements of the General Data Protection Regulation (GDPR) and Data Protection Act 2018. All files from data collection will be securely stored in locked cabinet. Electronic data will be initially stored in a password protected laptop and then transferred to one drive at earliest possibility to prevent loss of data. Only limited people directly involved in the study will have access to data. Data will be stored for a period of 10 years and then be safely destroyed. Hard copies having personal identifiers will be kept until entries of data and then will be securely destroyed. Other research related documents such as consent form will be scanned and securely destroyed.

***Plan for data analysis:***

Quantitative: Data will be managed using SPSS version 20.0. Initially, the demographic and baseline characteristics will be reported using descriptive statistics as frequencies and percentages, mean or median and standard deviation as appropriate. Initially, information collected at different intervals on primary and secondary outcomes will be reported through descriptive summary and comparison will be done using chi-square test for categorical and independent sample t-test for continuous data. The estimated mean difference between and within groups will be calculated along 95% confidence intervals with and statistical significance set at p<0.05. Cost effective analysis will be performed using an incremental cost-effectiveness ratio (ICER) that will measure the economic value of the intervention by comparing the incremental costs and health outcomes (e.g., QALYs gained, HbA1c reduction) between the intervention and control groups. Qualitative: Interviews will be audio recorded and then transcription in Nepali and subsequently translated to English for analysis. The qualitative data will be analysed using a thematic analysis approach (19). Interview transcripts will be read and re-read, formally coded and categorized and sub-categorized into emerging thematic areas. The results will be explained under the thematic areas and will be supported by verbatim quotes.

***Potential Biases***

There may be a chance of selection bias while assigning participants to intervention or control group. To minimize this, a simple randomization technique (Kang et al., 2008) will be used to assign participants to either intervention or control group on 1:1 basis. Concealment of allocation will be done using Sequentially numbered, opaque, sealed envelope (SNOSE) technique (Sil et al., 2019). An open-source random number sequence generator (random.org) will be used to generate random numbers in two columns (77 each) from 1 to 154 indicating column one for intervention group and column two for control group (random.org). The generated numbers in column one and column two will be kept in sequential order in two separate opaque sealed envelopes with a number labelled on outside. A statistician will generate the random number and prepare the envelopes while the research assistant will assign the participants to intervention or control group accordingly.

***Limitations of the study (if any):***

The study is limited to people who are able to use mobile phones therefore may owe to digital literacy bias. The intervention does not allow blinding of the participants and those assessing the outcomes.

***Plan for supervision and monitoring:***

Data collection will be closely monitored by the principal investigator. Data will be checked for completeness and correctness. Data Safety and Monitoring Committee has been formed to report any deviation or inconsistencies during the study.

***Plan for dissemination of the research:***

We will present the results in national and international conferences and publish the findings in the national and international peer-reviewed journals so that findings are accessible to a wide range of audiences.

**Data and Specimen Banking and/or sharing**

NA

**WORK PLAN**

| **S.N.** | **Activities** | **Timeline** |
| --- | --- | --- |
| 1. | Ethical approval | Oct 2024 |
| 2. | Trial registration | Nov 2024 |
| 3. | Formative study | Jan-Feb 2025 |
| 4. | Development of intervention | Mar-Jun 2025 |
| 5. | Recruitment and baseline data collection | Jul-Aug 2025 |
| 6. | Intervention delivery | Sept 2025-Feb 2026 |
| 7. | Follow up data collection | Mar-May 2026 |
| 8. | Data analysis | May-Nov 2026 |
| 9. | Thesis writing | Jul-Dec 2026 |

**ETHICAL CONSIDERATION**

**SIGNIFICANCE OF THE STUDY**

In the resource limited countries like Nepal, mhealth technology can be a cost-effective approach to deliver care and improve the health outcomes. There are studies demonstrating the feasibility, acceptability (Bhandari et al., 2022; Ni et al., 2020) and even effectiveness (Bhandari et al., 2022) of mhealth in improving the health outcomes for hypertension in Nepal (Bhandari et al., 2022; Ni et al., 2020). There is also existing evidence of mhealth as an effective means to communicate health related information and supporting positive behaviour change in a short period of time (Tuitui et al., 2022).

Despite availability of body of evidence on acceptability of mhealth in improving service utilization and health outcomes for chronic conditions, the application of behaviour change interventions through mhealth for self-management of diabetes is yet to be evaluated.

**BALANCE OF RISK AND BENEFIT**

**Risk of the study and Preventive and alleviative measures for risk**

| C2.1. Risk management: | |
| --- | --- |
| **Potential risks identified** | **Proposed measures to mitigate risks** |
| 1. Minimal risk (blood sample collection) | Trained lab person will collect blood sample following standard protocol |
| 2. |  |
| 3. |  |

**Benefits of the study**

There is no direct benefit as such however findings from the study help to inform development and effectiveness including acceptability of a text based mhealth intervention. If proven to be effective in improving self-management among people with diabetes, findings can be used to recommend to integrate in routine care to improve self-management for people with type 2 diabetes. Participants involved in the FGDs and IDI will be provided with be provided with expenses for the meal or travel for the day as per Nepalese rate as a form of appreciation of their time. The cost of HbA1c tests for baseline and follow up will be covered from the research.

**OBTAINING THE CONSENT:**

***How will the informed consent be obtained from the research participants?***

Written informed consent will be obtained from different category of the participants. Participants will be fully informed about their rights to withdraw from the study at any time without any penalty. A record will be maintained to keep track of withdrawal including the participant decision to use of data. Confidentiality and anonymity will be maintained throughout the process by using password protected computers and ensuring that all data is anonymised before analysis. Digital data will be kept for 10 years and securely destroyed.

***Who will obtain the consent from the study participants?***

Lead researcher or research assistant

***Is there anything being withheld from the research participants at the time the informed consent is being sought? Mention “YES” or “NO***

*No*

**Compensation for research participants**

✓ Yes, please provide details: For participants participating in FGDs and IDIs, expenses for either meal or travel will be provided on Nepalese rate for the day as a form of appreciation for the time provided. For participants enrolled in RCT, the cost of HbA1c test for baseline and follow-up will be covered from the research, however there will not be any direct form of compensation to these group of participants.

**APPENDIX**

**REFERENCES**

1. Diabetes [Internet]. 2021 [cited 30 March 2022]. Available from: https://www.who.int/news-room/fact-sheets/detail/diabetes.

2. IDF. Diabetes around the world in 2021. International Diabetes Federation, 2021.

3. Dhungana RR, Pandey AR, Shrestha N. Trends in the prevalence, awareness, treatment, and control of hypertension in Nepal between 2000 and 2025: a systematic review and meta-analysis. International journal of hypertension. 2021;2021.

4. Shrestha DB, Budhathoki P, Sedhai YR, Marahatta A, Lamichhane S, Nepal S, et al. Type 2 Diabetes Mellitus in Nepal from 2000 to 2020: A systematic review and meta-analysis. F1000Research. 2021;10.

5. International Diabetes Federation. Nepal Diabetes report 2000 — 2045. 2021.

6. Ghimire U, Shrestha N, Adhikari B, Mehata S, Pokharel Y, Mishra SR. Health system’s readiness to provide cardiovascular, diabetes and chronic respiratory disease related services in Nepal: analysis using 2015 health facility survey. BMC public health. 2020;20(1):1-15.

7. Gyawali B, Ferrario A, van Teijlingen E, Kallestrup P. Challenges in diabetes mellitus type 2 management in Nepal: a literature review. Global health action. 2016;9(1):31704.

8. Upreti SR, Lohani GR, Magtymova A, Dixit LP. Strengthening policy and governance to address the growing burden of diabetes in Nepal. WHO South-East Asia journal of public health. 2016;5(1):40.

9. Dhimal M, Bista B, Bhattarai S, Dixit LP, Hyder MKA, Agrawal N, et al. Report of Non Communicabl Disease Risk Factors:STEPS Survey Nepal 2019. Kathmandu, Nepal: 2020.

10. Huda MD, Rahman M, Rahman MM, Islam MJ, Haque SE, Mostofa MG. Readiness of health facilities and determinants to manage diabetes mellitus: evidence from the nationwide Service Provision Assessment survey of Afghanistan, Bangladesh and Nepal. BMJ Open. 2021;11(12):e054031.

11. Adhikari B, Pandey AR, Lamichhane B, KC SP, Joshi D, Regmi S, et al. Non-Communicable Disease Service Readiness in Nepal: A Further Analysis of Nepal Health Facility Survey-2021. medRxiv. 2023:2023.02. 07.23285512.

12. Powers MA, Bardsley J, Cypress M, Duker P, Funnell MM, Fischl AH, et al. Diabetes self-management education and support in type 2 diabetes: a joint position statement of the American Diabetes Association, the American Association of Diabetes Educators, and the Academy of Nutrition and Dietetics. The Diabetes Educator. 2017;43(1):40-53.

13. AADE. An effective model of diabetes care and education: revising the AADE7 Self-Care Behaviors®. The Diabetes Educator. 2020;46(2):139-60.

14. Panagioti M, Richardson G, Small N, Murray E, Rogers A, Kennedy A, et al. Self-management support interventions to reduce health care utilisation without compromising outcomes: a systematic review and meta-analysis. BMC health services research. 2014;14:1-14.

15. Sukartini T, Nursalam N, Pradipta RO, Ubudiyah M. Potential Methods to Improve Self-Management in Those with Type 2 Diabetes: A Narrative Review. International Journal of Endocrinology and Metabolism. 2023;21(1).

16. Kang H, Park H-A. A mobile app for hypertension management based on clinical practice guidelines: development and deployment. JMIR mHealth and uHealth. 2016;4(1):e4966.

17. Fadilah SZ, Susanti IA, Setyorini DY, Pradipta RO. Effectiveness of Mobile-Based Health Interventions for the Management of Hypertensive Patients: A Systematic Review. Jurnal Ners. 2020;15(2).

18. Johnston L, Zemanek J, Reeve MJ, Grills N. The evidence for using mHealth technologies for diabetes management in low-and middle-income countries. J Hosp Manag Health Policy. 2018;2(10.21037).

19. Kitsiou S, Paré G, Jaana M, Gerber B. Effectiveness of mHealth interventions for patients with diabetes: an overview of systematic reviews. PloS one. 2017;12(3):e0173160.

20. Mao Y, Lin W, Weng J, Chen G. The clinical outcomes and effectiveness of mHealth interventions for diabetes and hypertension: a systematic review and meta-analysis. medRxiv. 2020.

21. Lu X, Yang H, Xia X, Lu X, Lin J, Liu F, et al. Interactive mobile health intervention and blood pressure management in adults: a meta-analysis of randomized controlled trials. Hypertension. 2019;74(3):697-704.

22. Wang Y, Min J, Khuri J, Xue H, Xie B, Kaminsky LA, et al. Effectiveness of mobile health interventions on diabetes and obesity treatment and management: systematic review of systematic reviews. JMIR mHealth and uHealth. 2020;8(4):e15400.

23. Xiong S, Berkhouse H, Schooler M, Pu W, Sun A, Gong E, et al. Effectiveness of mHealth interventions in improving medication adherence among people with hypertension: a systematic review. Current hypertension reports. 2018;20(10):1-15.

24. CBS. National Population and Housing Census 2021. 2021.

25. Bhandari B, Narasimhan P, Jayasuriya R, Vaidya A, Schutte AE. Effectiveness and Acceptability of a Mobile Phone Text Messaging Intervention to Improve Blood Pressure Control (TEXT4BP) among Patients with Hypertension in Nepal: A Feasibility Randomised Controlled Trial. Global heart. 2022;17(1).

26. Ni Z, Atluri N, Shaw RJ, Tan J, Khan K, Merk H, et al. Evaluating the feasibility and acceptability of a mobile health–based female community health volunteer program for hypertension control in rural Nepal: cross-sectional study. JMIR mHealth and uHealth. 2020;8(3):e15419.

27. Tuitui RL, Bhatt A, Pradhan S, Hutchinson G, Gowland S, Saha S, et al. Using mobile health to strengthen the communication skills for effective delivery of health information in Nepal: A qualitative study of the perspectives of Female Community Health Volunteers. Journal of Global Health Economics and Policy. 2022;2:e2022008.

28. Chan A-W, Tetzlaff JM, Altman DG, Laupacis A, Gøtzsche PC, Krleža-Jerić K, et al. SPIRIT 2013 statement: defining standard protocol items for clinical trials. Annals of internal medicine. 2013;158(3):200-7.

29. Eysenbach G, Group C-E. CONSORT-EHEALTH: improving and standardizing evaluation reports of Web-based and mobile health interventions. Journal of medical Internet research. 2011;13(4):e1923.

30. Michie S, Van Stralen MM, West R. The behaviour change wheel: a new method for characterising and designing behaviour change interventions. Implementation science. 2011;6:1-12.

31. Fahim C, Acai A, McConnell MM, Wright FC, Sonnadara RR, Simunovic M. Use of the theoretical domains framework and behaviour change wheel to develop a novel intervention to improve the quality of multidisciplinary cancer conference decision-making. BMC health services research. 2020;20:1-19.

32. Fulton EA, Brown KE, Kwah KL, Wild S, editors. StopApp: using the behaviour change wheel to develop an app to increase uptake and attendance at NHS Stop Smoking Services. Healthcare; 2016: MDPI.

33. Gould GS, Bar-Zeev Y, Bovill M, Atkins L, Gruppetta M, Clarke MJ, et al. Designing an implementation intervention with the Behaviour Change Wheel for health provider smoking cessation care for Australian Indigenous pregnant women. Implementation science. 2017;12:1-14.

34. Munir F, Biddle SJ, Davies MJ, Dunstan D, Esliger D, Gray LJ, et al. Stand More AT Work (SMArT Work): using the behaviour change wheel to develop an intervention to reduce sitting time in the workplace. BMC public health. 2018;18:1-15.

35. Robinson H, Hill E, Direito A, Peel J, Saunders M, Ray M, et al. Use of the Behaviour Change Wheel to develop an intervention to promote physical activity following pulmonary rehabilitation in patients with COPD. 2019.

36. MacPherson MM, Cranston KD, Locke SR, Bourne JE, Jung ME. Using the behavior change wheel to develop text messages to promote diet and physical activity adherence following a diabetes prevention program. Translational Behavioral Medicine. 2021;11(8):1585-95.

37. Reidy C, Foster C, Rogers A. A facilitated web-based self-management tool for people with type 1 diabetes using an insulin pump: intervention development using the behavior change wheel and theoretical domains framework. Journal of Medical Internet Research. 2020;22(5):e13980.

38. Smith R, Michalopoulou M, Reid H, Riches SP, Wango Y, Kenworthy Y, et al. Applying the behaviour change wheel to develop a smartphone application ‘stay-active’to increase physical activity in women with gestational diabetes. BMC Pregnancy and Childbirth. 2022;22(1):253.

39. Xie LF, Roy-Fleming A, Haag S, Costa DD, Brazeau A-S. Development of the Support self-guided, web application for adults living with type 1 diabetes in Canada by a multi-disciplinary team using a people-oriented approach based on the Behaviour Change Wheel. Digital Health. 2023;9:20552076231152760.

40. Ritter PL, Lorig K, Laurent DD. Characteristics of the Spanish-and English-language self-efficacy to manage diabetes scales. The Diabetes Educator. 2016;42(2):167-77.

41. Kerari A. The psychometric properties of the Diabetes Self‐Efficacy Scale in Saudis with type 2 diabetes. Nursing Open. 2023;10(9):6408-15.

42. Mankan T, Erci B, Turan GB, Aktürk Ü. Turkish validity and reliability of the Diabetes Self-Efficacy Scale. International Journal of Nursing Sciences. 2017;4(3):239-43.

43. Dwa N, Panthee B. Perceived self-efficacy and self-care practices among diabetic patients in a Tertiary Hospital, Nepal. Journal of Diabetes and Endocrinology Association of Nepal. 2021;5(1):25-32.

44. Zimet GD, Dahlem NW, Zimet SG, Farley GK. The multidimensional scale of perceived social support. Journal of personality assessment. 1988;52(1):30-41.

45. Tonsing K, Zimet GD, Tse S. Assessing social support among South Asians: The multidimensional scale of perceived social support. Asian journal of psychiatry. 2012;5(2):164-8.

46. Akter J, Islam RM, Chowdhury HA, Selim S, Biswas A, Mozumder TA, et al. Psychometric validation of diabetes distress scale in Bangladeshi population. Scientific Reports. 2022;12(1):562.

47. WHOQOL User Manual [Internet]. 1998. Available from: https://iris.who.int/bitstream/handle/10665/77932/WHO_HIS_HSI_Rev.2012.03_eng.pdf?sequence=1.

48. Almarabheh A, Salah AB, Alghamdi M, Al Saleh A, Elbarbary A, Al Qashar A, et al. Validity and Reliability of the WHOQOL-BREF in the Measurement of the Quality of Life of Sickle Disease Patients in Bahrain. Frontiers in psychology. 2023;14:1219576.

49. Kalfoss MH, Reidunsdatter RJ, Klöckner CA, Nilsen M. Validation of the WHOQOL-Bref: psychometric properties and normative data for the Norwegian general population. Health and quality of life outcomes. 2021;19:1-12.

50. Sreedevi A, Cherkil S, Kuttikattu DS, Kamalamma L, Oldenburg B. Validation of WHOQOL-BREF in Malayalam and determinants of quality of life among people with type 2 diabetes in Kerala, India. Asia Pacific Journal of Public Health. 2016;28(1_suppl):62S-9S.

51. Joshi KD, Thapa J, Bhandary S. Assessing quality of life among older persons using WHOQOL-BREF tool–a pilot study in Chandragiri, Municipality: Quality of life among older persons. Journal of General Practice and Emergency Medicine of Nepal. 2023;10(15):18-23.

52. Toobert DJ, Hampson SE, Glasgow RE. The summary of diabetes self-care activities measure: results from 7 studies and a revised scale. Diabetes care. 2000;23(7):943-50.

53. Jalaludin M, Fuziah M, Hong J, Adam BM, Jamaiyah H. Reliability and validity of the revised summary of diabetes self-care activities (SDSCA) for Malaysian children and adolescents. Malaysian family physician: the official journal of the Academy of Family Physicians of Malaysia. 2012;7(2-3):10.

54. Dobson R, Whittaker R, Jiang Y, Shepherd M, Maddison R, Carter K, et al. Text message-based diabetes self-management support (SMS4BG): study protocol for a randomised controlled trial. Trials. 2016;17:1-10.

55. Dhimal M, Bista B, Bhattarai S, Dixit LP, Hyder MKA, Agrawal N, et al. Report of Non Communicable Disease Risk Factors: STEPS Survey Nepal 2019. Nepal Health Research Council, 2019.

56. Islam SMS, Niessen LW, Ferrari U, Ali L, Seissler J, Lechner A. Effects of mobile phone SMS to improve glycemic control among patients with type 2 diabetes in Bangladesh: a prospective, parallel-group, randomized controlled trial. Diabetes Care. 2015;38(8):e112-e3.

57. Kang M, Ragan BG, Park J-H. Issues in outcomes research: an overview of randomization techniques for clinical trials. Journal of athletic training. 2008;43(2):215-21.

58. Sil A, Kumar P, Kumar R, Das NK. Selection of control, randomization, blinding, and allocation concealment. Indian dermatology online journal. 2019;10(5):601-5.

59. Random sequence generator [Internet]. Available from: https://www.random.org/sequences/.
